# The Effect of Herbal Denture Adhesive on Patient Satisfaction in Comparison to Traditional Type

**DOI:** 10.7759/cureus.46001

**Published:** 2023-09-26

**Authors:** Maha Mekkawy, Ola Samy, Rahaf Alruhaimi

**Affiliations:** 1 Department of Prosthodontics, Qassim University, Qassim, SAU; 2 Dental Surgery, Qassim University, Qassim, SAU

**Keywords:** edentulous, retention, complete denture, satisfaction, denture adhesive

## Abstract

Background

The aim of this study was to compare the responses of 30 denture wearers’ opinions concerning the efficiency of two types of adhesives and estimate their satisfaction level using denture adhesive (DA).

Materials and methods

30 completely edentulous patients were divided randomly into two groups. Each group received one type of DAs (SECURE, COREGA) and was instructed to follow the same sequence of application. Each type of adhesive was used for seven days, as recommended by the manufacturer. The patients were requested to fill out a questionnaire considering their opinion and satisfaction regarding denture retention, chewing ability and duration of adhesives.

Results

The majority of patients stated that the prosthetic DAs enhanced their retention, chewing, stability, and efficiency of dentures. There was a significant difference in patient satisfaction regarding denture retention in the mandible and the duration of the adhesive in the mouth. Concerning ability of the patient to remove the adhesive from their mouth, the taste, or the retention of the maxillary denture, there were no statistically significant differences between the two DAs.

Conclusions

The results of the present study showed that overall satisfaction of complete denture wearers was high significantly different when a herbal DA was applied. This includes improvements in their duration of retention effectiveness, greater stability, and retention of the lower mandibular dentures. To use DA effectively, patients should be oriented by their dentists. Patient education is essential to provide them with a better understanding of both the appropriate technique for applying DA and the possible outcome they may experience.

## Introduction

Edentulism, described as an absence or complete loss of all-natural dentition, is a widespread phenomenon [1]. One of the most frequent issues with edentulous patients is severe atrophy of the alveolar ridge, which can happen despite careful prosthetic handling [2,3]. Therefore, the rehabilitation of fully edentulous patients is considered as one of the chief challenges in dentistry. Dentists used denture adhesive (DA) to improve denture adhesion and retention in order to deal with this problem [4].

According to studies, DAs, when properly applied, benefit complete denture patients with improved stability and efficiency by increasing the surface tension between denture fitting surface and alveolar mucosa [4,5]. DA should be secure, affordable, and have sufficient antibacterial and fungal characteristics. Additionally, it must have good taste and smell, it must be simple to use and access, and it must not alter or impair the denture's intaglio surface [2].

DA are now acknowledged as treatments that can be used in conjunction with dentures. As it may be challenging for elderly patients to physically and emotionally adapt to new or relined dentures, a DA can assist the patient in adjusting to the prosthesis's new shape, fit, and occlusion [6]. DA raises the patient's sensation of security and happiness. However, the application of DA must be preceded by consulting a dental professional.

There are many types of DA in the market. Some products are made from natural herbal materials such as aloe vera, which has been used in dentistry to treat oral mucosal lesions, gingivitis, and wounds, and to decrease plaque. Aloe vera can also speed up the healing process and lessen the pain resulting from oral ulcers [7]. Therefore, this study aims to evaluate patients' perceptions of herbal DA effectiveness in comparison to traditional type regarding denture retention, improvement of mastication, ease of use, speaking efficiency, improving self-confidence, and function. In addition to determine patients' satisfaction with this material and willingness to use it again.

## Materials and methods

This cross-sectional study, (approval number of a committee of ethics is 1442-635543) was conducted on 30 completely edentulous patients (17 male and 13 female, aged between 40 and 74 years) who received complete denture at Qassim University Dental Clinics and Qassim Regional Dental Center, Buraydah, KSA. According to the inclusion criteria, the following patients were selected: adult (40 years or older), mentally receptive patients seeking new complete dentures. Patients with masticatory dysfunction, difficulty responding to the questionnaires, and history of allergic reaction were excluded.

Study design

According to the DA type, the patients were randomly divided into equal two groups as follows: Group I, in which the patient used SECURE DA (Herbal); and Group II, COREGA DA (Table 1).

**Table 1:** Tested DA manufacturers and composition DA: Denture adhesive

Product	Manufacturer	Composition
Super COREGA	Stafford-Miller Ltd. Welwyn Garden City, Herts, UK	Calcium/Sodium PVM/MA Copolymer, Petrolatum, Cellulose Gum, Paraffinum Liquidum, Propylparaben, Aroma, Cl 45430. Does not contain Zinc.
SECURE	6 Grandinetti Drive, Ghent, NY 12075	Polyvinyl methyl ether-maleic acid (PVM-MA) copolymer alkali salt and sodium carboxymethyl cellulose (CMC). In addition, Aloe Vera Gel, Commiphora Myrrha Extract.

**Figure 1:**
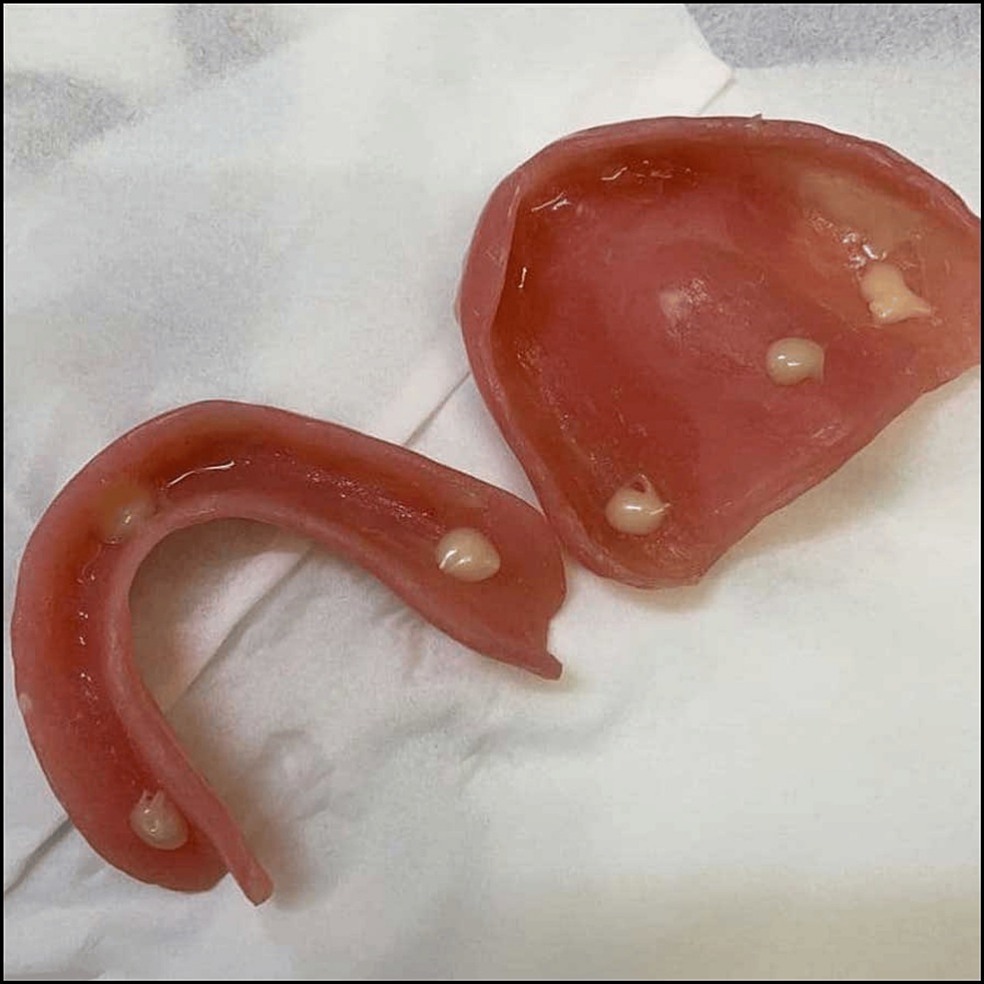
Maxillary and mandibular dentures showing the amount of DA to be applied in specific areas in the fitting surface DA: Denture adhesive

All patients were requested to fill out a questionnaire to include their opinion about retention, chewing, phonetic, removal, and fitting of denture (Table 2). After receiving the patient’s response on Questionnaire 1, clinical examination and demonstration on how to apply the DA were done following the manufacturer's instructions. The patients were informed about care instructions and cleaning for the next seven days (Figure 1). 

**Table 2:** Questionnaire 1 Modified from Kelsey et al. and Kulak et al. [8,9]

S.No.	Questionnaire	
1	How satisfied are you with the retention of your upper denture?	a. Very satisfied
		b. Fairly satisfied
		c. Not quite
		d. Dissatisfied
2	How satisfied are you with the retention of your lower denture?	a. Very satisfied
		b. Fairly satisfied
		c. Not quite
		d. Dissatisfied
3	Are you satisfied with the chewing ability of your denture?	a. Very satisfied
		b. Fairly satisfied
		c. Not quite
		d. Dissatisfied
4	Are you satisfied with the comfort of your new denture?	a. Very satisfied
		b. Fairly satisfied
		c. Not quite
		d. Dissatisfied
5	Does your denture cause any trauma to the soft tissues?	a. Yes
		b. No
6	Do you have any swallowing problems associated with the denture?	a. Yes
		b. No
7	How satisfied are you with the speech and the sound when using the denture?	a. Very satisfied
		b. Fairly satisfied
		c. Not quite
		d. Dissatisfied
8	How would you rate the removal and fitting of your dentures?	a. Very satisfied
		b. Fairly satisfied
		c. Not quite
		d. Dissatisfied

After one week, the patients were recalled to fill out another questionnaire to include their opinion about the strength, biocompatibility, convenience, and masticatory ability of the denture with the adhesive received (Table 3).

**Table 3:** Questionnaire 2 Modified from Kelsey et al. and Kulak et al. [8,9]

S.No.	Questionnaire	
1	How satisfied are you with the retention of your upper denture when using this adhesive?	a. Very satisfied
		b. Fairly satisfied
		c. Not quite
		d. Dissatisfied
2	How satisfied are you with the retention of your lower denture when using this adhesive?	a. Very satisfied
		b. Fairly satisfied
		c. Not quite
		d. Dissatisfied
3	Did the use of this denture adhesive have an effect on your ability to chew?	a. Much better
		b. Litter better
		c. No difference
		d. Worse
4	How long did this denture adhesive have an effect on your dentures?	a. ≤ 2 hours
		b. 2 – 4 hours
		c. 4 – 6 hours
		d. 6 – 12 hours
5	Did you like the taste of this denture adhesive?	a. Good
		b. Fairly good
		c. Worse
6	How was the removal of the adhesive from your dentures?	a. Easy
		b. Not easy
		c. Very difficult
7	Was it difficult to clean your denture after the denture adhesive had been applied?	a. Easy
		b. Not easy
		c. Very difficult
8	Was it difficult to clean your gums after the denture adhesive had been applied?	a. Easy
		b. Not easy
		c. Very difficult

Statistical analysis

Data were collected and analyzed using SPSS statistical software (version 23). Qualitative variables were presented using frequency percentage, while age was presented using mean and standard deviation. Age was compared between the two groups using independent t test, while all qualitative variables were compared using Pearson Chi Square test or Fisher’s exact test at a significant level of p value ≤0.05. The overall result satisfaction was calculated by summing the score of each questionnaire. Then, extracted the satisfaction percentage for each patient in both groups. Wilcoxon singed ranks test was performed to determine the difference in satisfaction among patients pre and post intervention.

## Results

In the present study, 30 patients (17 males and 13 females, aged between 40 and 74 years old; mean age 57.66 ± 9.85) were involved. The statistical analysis did not show significant differences between the rate of satisfaction regarding the age and gender factors (p=0.691, p=0.269). The results are shown in Table 4. The frequency of distribution of the patients' responses is shown in Table 5.

**Table 4:** Demographic data of the study patients

		SECURE Adhesive (n=15)	COREGA Adhesive (n=15)	Test (p value)	Total (n=30)
Age: Mean (SD)		58.40 (8.72)	56.93 (11.13)	0.402 (0.691)	57.66 (9.85)
Gender: n (%)	Males	10 (66.7%)	7 (46.7%)	1.222 (0.269)	17 (56.7%)
	Females	5 (33.3%)	8 (53.3%)		13 (43.3%)

**Table 5:** The frequency of patients' responses *Statistically significant difference at (p value ≤0.05). DA: Denture adhesive

Responses after Adhesives		SECURE Adhesive (n=15)	COREGA Adhesive (n=15)	Test (p value)
		n (%)		
Satisfaction with retention of the upper denture	Dissatisfied	0 (0%)	0 (0%)	1.429 (0.427)
	Not quite	0 (0%)	0 (0%)	
	Fairly satisfied	6 (40%)	3 (20%)	
	Very satisfied	9 (60%)	12 (80%)	
Satisfaction with retention of lower the denture	Dissatisfied	0 (0%)	0 (0%)	7.046 (0.030*)
	Not quite	1 (6.7%)	5 (33.3%)	
	Fairly satisfied	1 (6.7%)	4 (26.7%)	
	Very satisfied	13 (86.7%)	6 (40%)	
DA effect on ability to chew	No difference	2 (13.3%)	1 (6.7%)	2.807 (0.246)
	Little better	2 (13.3%)	6 (40%)	
	Much better	11 (73.3%)	8 (53.3%)	
Duration of adhesive effect on the denture	≤2 hours	0 (0%)	0 (0%)	8.674 (0.013*)
	2–4 hours	1 (6.7%)	2 (13.3%)	
	4–6 hours	3 (20%)	10 (66.7%)	
	6–12 hours	11 (73.3%)	3 (20%)	
Taste of the adhesive	Worse	0 (0%)	2 (13.3%)	3.843 (0.146)
	Fairly good	4 (26.7%)	1 (6.7%)	
	Good	11 (73.3%)	12 (80%)	
Removal of the adhesive	Very difficult	1 (6.7%)	0 (0%)	3.333 (0.189)
	Not Easy	2 (13.3%)	0 (0%)	
	Easy	12 (80%)	15 (100%)	
Cleaning the DA after application	Very difficult	0 (0%)	0 (0%)	2.727 (0.215)
	Not Easy	6 (40%)	2 (13.3%)	
	Easy	9 (60%)	13 (86.7%)	
Cleaning gums after adhesive application	Very difficult	1 (6.7%)	3 (20%)	1.200 (0.549)
	Not Easy	3 (20%)	3 (20%)	
	Easy	11 (73.3%)	9 (60%)	

Retention

For maxillary dentures, among the 30 patients, 12 patients (80%) and nine patients (60%) reported very satisfactory retention of maxillary dentures when placing COREGA and SECURE adhesives respectively. Statistically, no significant difference was found between the two adhesive types regarding maxillary denture retention (p=0.427).

For mandibular dentures, SECURE (86.7%) had higher denture retention satisfaction than COREGA (40%). A statistically significant difference was found between the two adhesive types regarding mandibular denture retention (SECURE: 13 and COREGA: 6; p=0.030).

Chewing ability

Regarding chewing effectiveness, 11 patients (73.3%) and eight patients (53.3%) reported that they were able to chew more effectively when they using SECURE and COREGA adhesives, respectively. Two patients (13.3%) reported a little better chewing ability with SECURE-based DA whereas six patients (40%) with COREGA. Chewing ability was not statistically significantly different between the two types of DA (p =0.246).

Duration of retention

The duration of retention efficiency in the mouth showed statistically significant difference between the two adhesives (SECURE: 11 and COREGA: 3; p=0.013). The SECURE adhesive was reported as effective by 11 patients (73.3%) for 12 hours, while the COREGA adhesive was reported as effective by 10 patients (66.7%) for four to six hours.

Taste

Taste properties of the two DAs were not significantly different (p=0.146).11 patients (73.3%) and 12 patients (80%) rated the taste of SECURE and COREGA respectively as good. Whereas, four (26.7%) patients reported that they found the taste were fairly good with SECURE, while one (6.7%) was fairly good with COREGA.

Removal

SECURE adhesive paste was easily removed by 12 patients (80%), while two patients (13.3%) found it difficult to accomplish. On the other hand, among 15 patients the removal of COREGA adhesive paste was found to be easy (100%). No significant differences were found between the two adhesive types concerning removal (p=0.189)

Cleaning the denture

13 (86.7%) and nine (60%) of the 30 patients found the cleaning of the denture when using COREGA and SECURE adhesive pastes easy to accomplish, respectively. A statistically significant difference was not found between adhesive types on cleaning the denture after application (p=0.215).

Cleaning the gum

Cleaning gums after adhesives application was found easy by 11 patients (73.3%) and nine patients (60%) when using SECURE and COREGA, respectively. In comparison, one patient (6.7%) and three patients (23%) found it very difficult to accomplish. Cleaning the gum with the two adhesives did not show statistically significant differences (p = 0.549).

**Figure 2:**
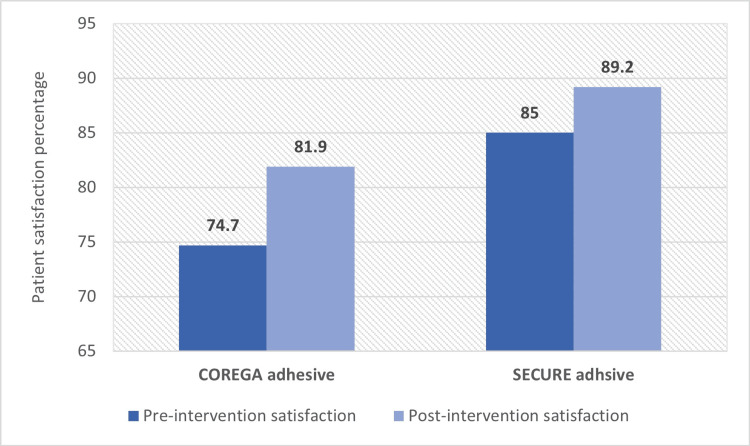
Comparison of overall satisfaction pre and post intervention

Table 6 shows that three patients in SECURE adhesive group had the same satisfaction percentage pre and post intervention. Nine patients had higher satisfaction score post intervention and three patients had higher satisfaction pre intervention. There was no significant difference in patients’ satisfaction pre and post intervention where p-value was 0.182. Affect size was calculated as

$$r = \frac{z}{\sqrt{N}} = \frac{-1.334b}{\sqrt{30}}=-0.243$$,

which consider as a small affect size. While in COREGA group, six patients had higher satisfaction score pre intervention than post intervention, nine patients had a higher satisfaction score in post intervention. There was no significant difference in patients’ satisfaction pre and post intervention where p-value was 0.191. The effect size was -0.238, which is a small effect size. The average satisfaction percentage for each group is shown in Figure 2.

**Table 6:** Wilcoxon singed ranks test for the SECURE and COREGA adhesive groups SP1 = Satisfaction percentage pre intervention*; *SP2 = Satisfaction percentage post intervention* *

		SECURE				COREGA			
		N	Mean Rank	Sum of Ranks	P (Wilcoxon Test)	N	Mean Rank	Sum of Ranks	P (Wilcoxon Test)
SP2 - SP1	Negative Ranks	3	7.33	22.00	0.182	6	6.17	37.00	0.191
	Positive Ranks	9	6.22	56.00		9	9.22	83.00	
	Ties	3				0			
	Total	15				15			

 

In COREGA adhesive group, the average satisfaction percentage was 74.7%. After the intervention, it increased to 81.9%. In SECURE adhesive GROUP, the average satisfaction score was 85%, and it increased to 89.2% after the intervention.

## Discussion

The success of complete denture treatment is a result of a number of reasons, including technical excellence, appropriate patient education, and the dentist's awareness of all treatment techniques in order to accomplish the maximum patient satisfaction [10]. In the past, dentists thought the use of denture retainers indicated poor dentist skills, or thought as a solution for an ill-fitting denture. However, this perspective has changed [2]. For patients who need extra retention that cannot be met by the standard protocol of complete denture construction, the use of denture adhesion is highly recommended [11]. Commercial DAs are the materials that can enhance patient satisfaction and the quality of care when appropriately used. 

The present study focused on the impact of adhesives on the retention of complete dentures, adhesive strength, patient convenience, masticatory ability, and biocompatibility that was reported subjectively in order to determine whether herbal DA provided a better level of patient satisfaction in comparison to traditional type.

The results of this study are in agreement with the participants’ baseline characteristic with other studies that have shown no significant difference in age and gender when DA was used [2,12]. In this study, the majority of patients reported that the prosthetic DAs increased retention, stability, and incisive ability of prosthesis. In addition, patients reported that their dentures were also more comfortable. These findings are in consistent with the previous studies [4,13,14].

When using the COREGA and SECURE adhesive pastes for the maxillary dentures, the majority of patients in this study were either very satisfied (80%), (60%) or fairly satisfied (20%), (40%). No statistically significant differences were found for retention of maxillary dentures. This finding can be explained as mentioned by Kulak et al., in which the maxillary dentures engage a greater quantity of space, and ridges are often less resorbed in comparison to the highly resorbed alveolar ridges in the mandible [9].

The retention of mandibular dentures is difficult to achieve. The reason could be due to severe residual ridge resorption in the mandible, as well as the lack of a palatal vault, and a smaller denture-bearing surface on the mandibular ridge. Therefore, the stress on the lower ridge is greater than that on the upper ridge [15,16]. The statistically significant difference was observed between the two adhesive types on mandibular dentures. These findings are in line with those of El-Mekawy et al. and Kulak et al. [4,9].

The study rated chewing ability from a little better to much better. This is consistent with the reports of Neil and Roberts [17] who found that the use of DAs significantly improved chewing ability in subjects with poorly or moderately fitting dentures dentures. This finding is along with the previous studies [18,19]. Despite this finding, El-Mekawy et al. and Hakeem et al. showed no statistically significant difference in the improvement of chewing between the two groups of adhesives, whereas the study of Kulak et al. and Koronis et al. showed that a statistically significant difference in chewing ability was reported [4,6,9,10]. 

According to the present result of this study, concerning the removal of adhesive from fitting surfaces of dentures, most of the patients found that DAs were easily removed, and this finding agrees with Koronis et al. [6]. One important finding of this study is that there is a significantly higher herbal DA effect on retention duration compared to the traditional type. The properties of an adhesive composition play a major role in determining the retention effect of the adhesive (Table 1). Herbal DA may have a longer duration due to its ingredients, such as acemannan, a polysaccharide derived from aloe vera gel, and due to its consistency, since gel is sticky and viscous. Previous studies developed a prototype acemannan DA, which demonstrated excellent adhesive strength both wet and dry. Compared to commercially available adhesive formulations, acemannan generally performs better in vitro, indicating that complex carbohydrates may be effective DAs [20,21].

In regard of the comparison between the pre and post intervention within groups as for overall satisfaction, there was no statistically significant difference. Nonetheless, the average satisfaction showed an increase in post-intervention, this finding is in agreement with Ohwada et al. that interpreted that the using of DA may have a reasonable impact on sensation perceived (Figure 2) [12]. Based on the results of the present study, DA significantly improved patient subjective satisfaction with their complete denture, taking in consideration that the use of adhesives should not be used for hiding clinical and laboratory imprecision as advised by Bogucki et al. [22].

One of the limitation of the current study in that it was conducting on a small group. Therefore, studies on a larger sample size are recommended in order to determine which type of DA is most effective.

## Conclusions

The results of the present study showed that overall satisfaction of complete denture wearers was high significantly different when a herbal DA was applied. This includes improvements in their duration of retention effectiveness, greater stability, and retention of the lower mandibular dentures. To use DA effectively, patients should be oriented by their dentists. Patient education is essential to provide them with a better understanding of both the appropriate technique for applying DA and the possible outcome they may experience.
